# Fault Diagnosis Method for Vacuum Contactor Based on Time-Frequency Graph Optimization Technique and ShuffleNetV2

**DOI:** 10.3390/s24196274

**Published:** 2024-09-27

**Authors:** Haiying Li, Qinyang Wang, Jiancheng Song

**Affiliations:** 1 School of Mechanical Engineering, University of Shanghai for Science and Technology, Shanghai 200093, China; sh_lhy@163.com; 2 Shanxi Key Laboratory of Mining Electrical Equipment and Intelligent Control, Taiyuan University of Technology, Taiyuan 030024, China; sjc6018@163.com

**Keywords:** vacuum contactor, vibration signal, time-frequency graph optimization, ShuffleNetV2 network, fault diagnosis

## Abstract

This paper presents a fault diagnosis method for a vacuum contactor using the generalized Stockwell transform (GST) of vibration signals. The objective is to solve the problem of low diagnostic performance efficiency caused by the inadequate feature extraction capability and the redundant pixels in the graph background. The proposed method is based on the time-frequency graph optimization technique and ShuffleNetV2 network. Firstly, vibration signals in different states are collected and converted into GST time-frequency graphs. Secondly, multi-resolution GST time-frequency graphs are generated to cover signal characteristics in all frequency bands by adjusting the GST Gaussian window width factor *λ*. The OTSU algorithm is then combined to crop the energy concentration area, and the size of these time-frequency graphs is optimized by 68.86%. Finally, considering the advantages of the channel split and channel shuffle methods, the ShuffleNetV2 network is adopted to improve the feature learning ability and identify fault categories. In this paper, the CKJ5-400/1140 vacuum contactor is taken as the test object. The fault recognition accuracy reaches 99.74%, and the single iteration time of model training is reduced by 19.42%.

## 1. Introduction

With the growth of transparency requirements for distribution networks, the transparency level of distribution networks is deepening, and the intellectualization upgrade for distribution terminals is accelerated [1,2]. The vacuum contactor, as one type of critical transparent management equipment in distribution networks, is used to frequently start and stop high-power load devices [3]. Its operational state is directly related to the reliability of the power grid. Vibration signals during the opening and closing are generated by severe collisions among components, containing rich information about the equipment health state. Therefore, these signals can be used to identify various mechanical faults, such as spring fatigue and iron core rusting [4].

Fault diagnosis based on vibration signals is divided into two parts: feature extraction and fault identification. A summary of the latest methods is shown in Table 1. In the feature extraction stage, vibration signal data are commonly processed using time-domain, frequency-domain, and time-frequency domain methods [5]. Time-domain analysis utilizes multiple parameter indexes to characterize the dynamic information. Typical time-domain features include skewness, impulse factor, shape factor, and various characteristic entropy [6,7,8]. Due to the fact that the time-domain features are not apparent during the early stages of equipment failure, fault identification is a challenge [9]. For this reason, frequency-domain analysis is adopted to convert time-series signals into an intuitive frequency spectrum. By extracting critical statistical features such as envelope spectrum [10], power spectrum [11], and cepstrum [12], the fault characteristics of vibration signals are enhanced or separated. However, frequency-domain analysis fails to capture the characteristics of transient vibration signals through Fourier transform [13].

Unlike time-domain or frequency-domain analyses that extract partial features of the signal, time-frequency domain analysis converts one-dimensional vibration signals into two-dimensional time-frequency graphs, fully reflecting the distribution of vibration signal characteristics [14]. Common time-frequency domain analysis methods include short-time Fourier transform (STFT), continuous wavelet transform (CWT), and Stockwell transform (ST). Ref. [15] employs STFT to convert vibration signals into time-frequency matrices. After normalization, the STFT time-frequency graphs are generated with comprehensive fault features. Refs. [16,17] convert vibration signals of the circuit breaker into CWT time-frequency graphs according to the selected scale and wavelet basis function, extracting the features in different states. Both of the time-frequency analysis methods can extract rich fault feature components. However, STFT uses a fixed window function, resulting in uneven time-frequency resolution and an inability to capture instantaneous frequency variations [18]. CWT, on the other hand, relies on fixed wavelet bases and suffers from issues such as frequency band energy leakage [19], leading to poor performance in high-frequency abrupt change scenarios. ST overcomes the above shortcomings by maintaining the phase information of the vibration signals and using different Gaussian window functions for each frequency band, providing good time-frequency resolution [20,21]. To obtain more time-frequency resolution datasets, the GST adjusts the size of the Gaussian window function by introducing the window width adjustment factor. The transient changes are easily captured, and all information across different frequency bands is represented [22]. Therefore, GST is more suitable for feature extraction to various transient signals.

**Table 1:** A summary of the latest methods in fault diagnosis.

Field	Category	Reference	Method	Limitation
Feature extraction	Time-domain	[6]	Skewness, impulse factor	Not apparent during the early stages of equipment failure
		[7]	Shape factor	
		[8]	Characteristic entropy	
	Frequency-domain	[10]	Envelope spectrum	Fail to capture the features of transient signals through Fourier transform
		[11]	Power spectrum	
		[12]	Cepstrum	
	Time-frequency domain	[15]	STFT	Feature extraction with fixed window functions or wavelet bases
		[16,17]	CWT	
		[20,21]	ST	
Fault identification	Machine learning	[23,24]	BPNN	Poor-fitting performance for complex data
		[25]	SVM	
		[26,27]	RF	
	Deep learning	[28]	AlexNet	Constrained feature learning capability due to the limited convolutional and pooling layers
		[29]	ResNet50	The number of parameters grows as the layers deepen
		[30]	ResNeXt50	More computational resources are occupied due to the excessive group convolution

For fault identification, the traditional diagnostic algorithms include back propagation neural network (BPNN) [23,24], support vector machine (SVM) [25], and random forest (RF) [26,27]. Compared with the machine learning algorithms above, deep learning, AlexNet [28] as an example, can automatically extract features from target data without expert knowledge, showing significant advantages in image processing tasks. To extract features fully, as advanced networks, ResNet50 [29] and ResNeXt50 [30] incorporate residual blocks to keep deep networks. For higher accuracy, ResNeXt50 introduces group convolution, dividing each residual block into multiple branches. However, massive parameters in deep learning networks and excessive group convolutions occupy more computational resources during the training of ResNet50 and ResNeXt50 models [31,32]. ShuffleNetV2, combined with channel split and channel shuffle operations, is designed with full consideration of memory access cost, greatly enhancing computational efficiency [33]. It guarantees the balance between computational complexity and recognition accuracy. In recent years, ShuffleNetV2 has begun to draw attention to equipment fault identification [34].

Fault diagnosis at present still shows some limitations. For instance, the features in the dataset are insufficient, and the redundant background pixels of time-frequency graphs occupy computational resources. Moreover, complex neural networks generally have stronger fitting ability, but deep learning models may fail to be trained due to gradient explosion or gradient disappearance during training.

In response to the above issues, we propose a fault diagnosis method for vacuum contactors integrating the time-frequency graph optimization technique with ShuffleNetV2. The main contributions of this paper are summarized as follows:

(1) A data augmentation technique using multiple-resolution GST time-frequency graphs can fully extract the signal features of each frequency band.
(2) The OTSU algorithm is combined to crop key feature areas of the time-frequency graphs, removing redundant background pixels to improve training efficiency.
(3) The ShuffleNetV2 network is employed to construct the fault diagnosis model. The recognition accuracy and the training time are improved due to its lightweight network architecture.

The rest of this paper is organized as follows: Section 2 introduces the time-frequency graph of vibration signals. Section 3 presents the graph optimization technique in detail. Section 4 describes the principles of the ShuffleNetV2 fault diagnosis model. Section 5 discusses the optimization and diagnosis results. The research conclusions and directions for future work are provided in Section 6.

Considering the convenience of readers, the Abbreviations table lists the abbreviations used in this paper and their meanings.

## 2. Time-Frequency Graph of Vibration Signal

### 2.1. Vibration Signal Acquisition

**Figure 1:**
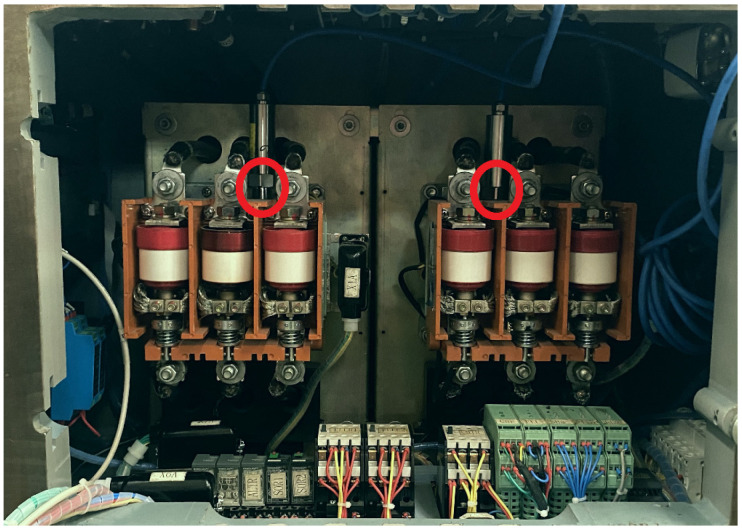
Acceleration sensor mounting position diagram.

The vacuum contactor vibration signal acquisition system consists of an acceleration sensor, a constant current power module, and a data acquisition card. Vibration signals are collected using a CT1000L piezoelectric acceleration sensor, with a measurement range from 0 to 1000 g and a sensitivity of 5 mV/g. This sensor is characterized by high sensitivity and excellent anti-interference performance. To enhance the reliability of the vibration signal, the acceleration sensor is fixed near the moving contacts, with its installation axis aligned with the vibration direction, as shown in Figure 1.

The opening and closing actions of the vacuum contactor accompany the vibration signals. Compared with the opening action, the closing action, with more vibration sources and longer vibration durations, is more suitable as an indicator of the health state. Therefore, closing vibration signals are collected to identify various mechanical faults.

This paper focuses on the CKJ5-400/1140 vacuum contactor, which can perform up to 2000 operations per hour and withstand transient current surges up to 10 times its rated operating conditions. It operates year-round in humid environments with an air humidity of approximately 90% [35]. The harsh working environment and frequent overcurrent surges often lead to various gradual failures.

Based on the working environment and typical fault types, three fault states are simulated by referencing the literature [36]. Closing vibration signals are collected at different times to increase the diversity of sample data. The specific fault simulation scheme is shown in Table 2.

**Figure 2:**
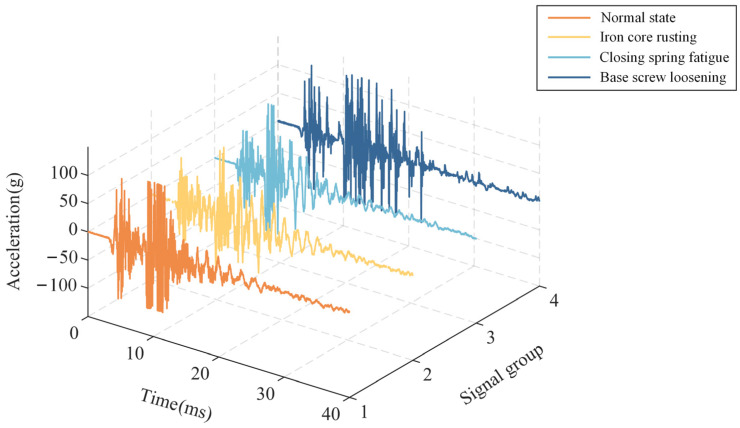
Closing vibration signal waveforms in different states.

To improve the noise rate by keeping high-frequency noise, the signal sampling frequency is set to 100 kHz for 40 ms. All vibration signals are recorded since the closing instruction. A set of closing vibration signals is randomly selected for each operating state, and the corresponding waveform is shown in Figure 2.

As shown in Figure 2, the vibration signals exhibit transient and non-stationary characteristics in different states with the following features:

(1) Normal state: The high-energy vibration is induced by the energized core and the closing main contacts, resulting in the primary and secondary peak. The signal energy gradually diminishes 20 ms later.
(2) Iron core rusting fault: When this fault occurs, rust and debris increase resistance to movement, weakening the vibration signal energy. The amplitudes in the primary peak and secondary peak are slightly lower than those in the normal state.
(3) Closing spring fatigue fault: When this fault occurs, the mechanical properties of the spring degrade and its elasticity declines. Compared with the core rusting fault and normal state, the closing action is easier to approach stability. Therefore, the vibration occurs earlier, and the primary peak is short.
(4) Base screw loosening fault: the system’s damping diminishes in this case, which leads to an increase in the primary peak and secondary peak, consuming longer time for the signal to decay.

### 2.2. GST Time-Frequency Graph

The closing vibration signals undergo rapid changes within a very short period. It is difficult to characterize their transient frequency-domain characteristics with traditional time-frequency analysis methods. The Stockwell transform introduces the Gaussian window function, which converts one-dimensional time-series signals into two-dimensional time-frequency matrices. This method provides all local features in the time-frequency graph. The Stockwell transform of the closing vibration signal *x*(*t*) of a vacuum contactor is given as follows:

(1) $$S(\tau ,f)=\int_{-\infty }^{\infty }x(t)\omega (\tau -t,f)e^{-i2\pi ft}dt$$

where *t* is time, *τ* is the time-shift factor, *f* is continuous frequency, i is the imaginary unit, and *ω*(*τ* − *t*, *f*) is the Gaussian window function, defined as follows:

(2) $$\omega (\tau -t,f)=\frac{\left|f\right|}{\sqrt{2\pi }}e^{-\frac{{\left(t-\tau \right)}^{2}f^{2}}{2}}$$

From Formula (2), it is evident that the size of the Gaussian window function changes at a fixed rate with frequency. When the signal frequency changes rapidly within a certain period, the Stockwell transform may fail to fully capture the detailed characteristics of the signal in that period. To improve time-frequency resolution, the GST introduces an adjustment factor *λ* into the Gaussian window function, allowing flexible adjustment of the window size. This enables the local characteristics of vibration signals in different states to be fully reflected in the time-frequency graph. The GST of the closing vibration signal *x*(*t*) is given as follows:

(3) $$S_{GST}(\tau ,f)=\int_{-\infty }^{\infty }x(t)\frac{\left|\lambda \right|\left|f\right|}{\sqrt{2\pi }}e^{-\frac{\lambda^{2}{\left(t-\tau \right)}^{2}f^{2}}{2}}e^{-i2\pi ft}d\tau$$

**Figure 3:**
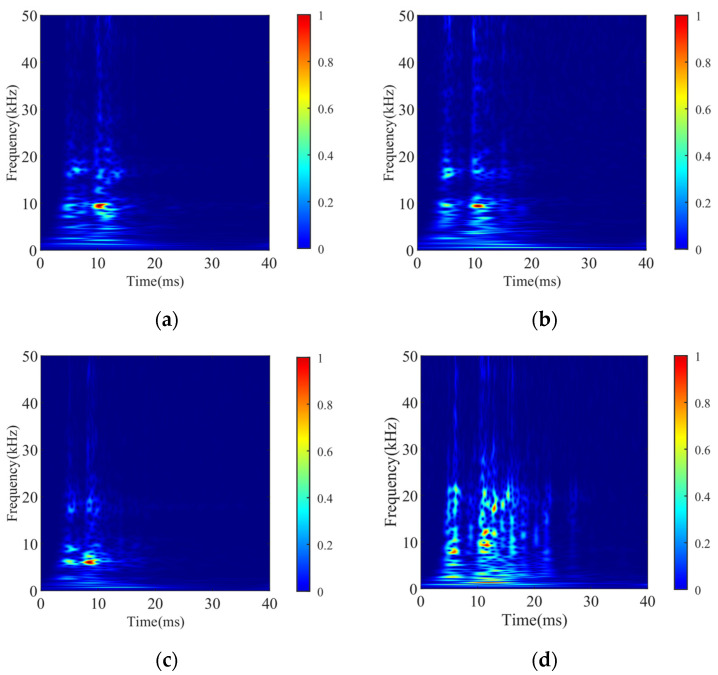
GST time-frequency graphs of vibration signals in different states: (**a**) normal; (**b**) iron core rusting; (**c**) closing spring fatigue; (**d**) base screw loosening.

When the Gaussian window width adjustment factor *λ* = 1, Formula (3) becomes the standard Stockwell transform. The adjustment factor *λ* will be further discussed in Section 3.1; for now, *λ* is set to 0.6. A closing vibration signal is randomly selected for each state, and the complex time-frequency matrix of the vibration signal is obtained using the discrete expression of the GST. After being taken modulus and normalized, the corresponding GST time-frequency graphs of the four states are shown in Figure 3.

## 3. Time-Frequency Graph Optimization Technique

### 3.1. Data Augmentation of Time-Frequency Graph

The GST adjustment factor *λ* affects the resolution of vibration signal time-frequency graphs. To extract detailed characteristics in various frequency bands and enhance the diversity of the time-frequency graph dataset, data augmentation optimization is performed by configuring multiple sets of adjustment factors.

According to the characteristics of the adjustment factor *λ*, when 0 < *λ* ≤ 1, the width of the Gaussian window decreases inversely with frequency at a decelerating rate, facilitating the extraction of features in the low-frequency range. Conversely, when *λ* > 1, the window width increases proportionally with frequency at an accelerating rate, making it suitable for extracting features in the high-frequency range [37,38]. A series of *λ* parameters are set to generate multiple GST time-frequency graphs with different resolutions from the same signal.

**Figure 4:**
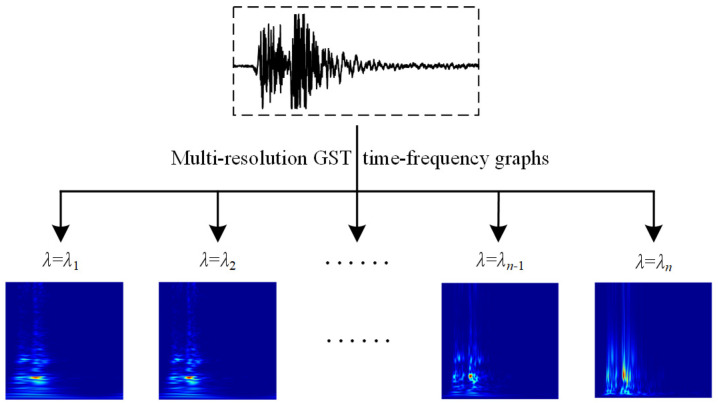
Vibration signal GST time-frequency graph data augmentation.

The time-frequency graph of the vibration signal corresponding to *λ* = 1 serves as the original data. A series of adjustment factors *λ*, denoted as *λ*_1_, *λ*_2_, …, *λ_n_*_−1_, and *λ_n_*, are evenly set. Each *λ* value is applied in the GST expression to generate multi-resolution GST time-frequency graphs, thereby realizing data augmentation, as shown in Figure 4.

From Figure 4, it is observed that after the vibration signal undergoes time-frequency transformations at different scales, the number of samples is expanded to *n* times that of the original data. This augmentation not only enriches the diversity of time-frequency graph samples but also characterizes the time-frequency distribution characteristics in both high- and low-frequency ranges.

### 3.2. Cropping Optimization of Time-Frequency Graph

The data augmentation technique enriches the time-frequency graph samples. However, non-peak regions in the time-frequency graphs lack effective characteristics, and the redundant pixels in the background occupy more computational resources. To improve model training and fault diagnosis efficiency, the OTSU algorithm is employed to crop energy-concentrated regions in the time-frequency graphs [39].

The OTSU algorithm, also known as the maximum between-class variance method, partitions an image into foreground and background based on an optimal threshold determined by the grayscale levels. It intends to ensure the between-class variance between foreground and background is maximized. Assuming the GST time-frequency graph contains *k* grayscale levels [0, 1, …, *k* − 1], if there exists a threshold *r* to divide the pixels of the time-frequency graph into two parts—the set *r***_1_** consists of pixels with grayscale values less than *r*, representing the background of the time-frequency graph, while the set *r***_2_** consists of pixels with grayscale values greater than *r*, representing the foreground of the time-frequency graph—then the probabilities of occurrence of *r***_1_** and *r***_2_**, denoted as *P*_1_ and *P*_2_, respectively, are as follows:

(4) $$\left\{\begin{array}{l} P_{1}=\sum_{j=0}^{r}\frac{n_{j}}{N}=\sum_{j=0}^{r}p_{j}\\ P_{2}=\sum_{j=r+1}^{k-1}\frac{n_{j}}{N}=\sum_{j=r+1}^{k-1}p_{j}=1-P_{1} \end{array}\right.$$

where *n_j_* is the number of pixels with grayscale *j*, *N* is the total number of pixels in the graph, and *p_j_* is the probability of pixels with grayscale *j* appearing in the graph.

The grayscale mean of the background, the grayscale mean of the foreground, and the entire image are denoted as *μ*_1_, *μ*_2_, and *μ*. The relationship among them is as follows:

(5) $$\mu =P_{1}\mu_{1}+P_{2}\mu_{2}$$

According to the definition of variance, the expression for the between-class variance *σ*^2^ is

(6) $$\sigma^{2}=P_{1}{\left(\mu_{1}-\mu \right)}^{2}+P_{2}{\left(\mu_{2}-\mu \right)}^{2}$$

Substituting Formulas (4) and (5) into Formula (6), the variational equation to maximize the between-class variance *σ*^2^ is constructed as follows:

(7) $$\left\{\begin{array}{l} \underset{\left\{r\right\}}{\max}\;\;\sigma^{2}=\sum_{j=0}^{r}p_{j}\sum_{j=r+1}^{k-1}p_{j}\cdot {\left(\mu_{1}-\mu_{2}\right)}^{2}\\ s.t.\;\;\;\;\sum_{j=0}^{k-1}p_{j}=1 \end{array}\right.$$

By traversing grayscale levels, the optimal threshold *r_op_* is determined by Formula (7). Pixels in the GST time-frequency graph below *r_op_* are set to zero, while those above *r_op_* are retained. The energy-concentrated portions are cropped based on the boundaries of non-zero pixels.

An arbitrary sample *i* from the GST time-frequency graph dataset is selected. Following the above steps of the OTSU algorithm, the time and frequency values corresponding to the non-zero pixel boundary points are denoted as $t_{\min}^{i}$, $t_{\max}^{i}$, $f_{\min}^{i}$, and $f_{\max}^{i}$, respectively. The energy concentration regions are located in the time interval [$t_{\min}^{i}$, $t_{\max}^{i}$] and frequency range [$f_{\min}^{i}$, $f_{\max}^{i}$].

**Figure 5:**
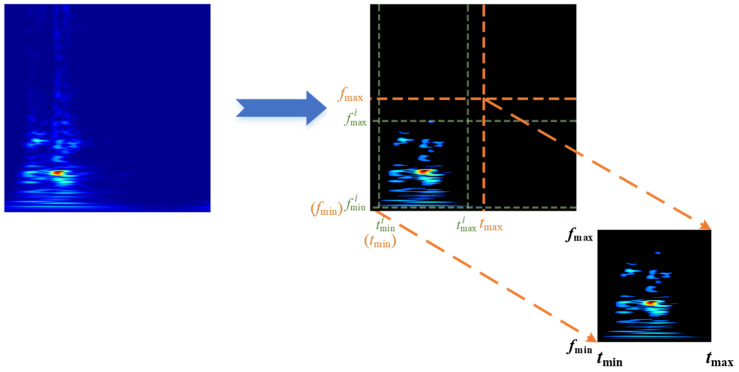
Cropping optimization of the GST time-frequency graph.

All the multi-resolution GST time-frequency graphs are traversed. The minimum values, *t*_min_ and *f*_min_, are obtained as min{$t_{\min}^{i}$, $f_{\min}^{i}$}, and the maximum values, *t*_max_ and *f*_max_, are obtained as max{$t_{\max}^{i}$, $f_{\max}^{i}$}. The GST time-frequency graphs are uniformly cropped to extract concentrated regions of feature distributions for various closing vibration signals, as depicted in Figure 5.

Figure 5 illustrates that the cropping optimization technique, combined with the OTSU algorithm, can remove redundant pixels while preserving the effective feature information of the critical frequency bands. It reduces the dimension of the GST time-frequency graph and retains the concentrated energy distribution regions of GST time-frequency graphs for different states.

## 4. ShuffleNetV2 Fault Diagnosis

### 4.1. Principles of ShuffleNetV2 Network

**Figure 6:**
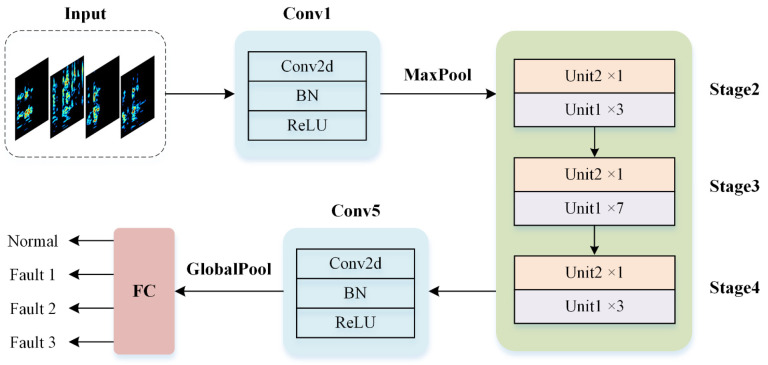
ShuffleNetV2 network architecture.

ShuffleNet is a lightweight convolutional neural network suitable for online fault diagnosis in edge computing scenarios. ShuffleNetV1 reduces the number of network parameters by introducing group convolutions. However, when the number of groups increases, the speed of inference decreases, restricting its applications in practice [40]. To enhance parallel computing efficiency and reduce memory access costs, ShuffleNetV2 utilizes channel split and channel shuffle operations to shorten network runtime and improve feature learning capabilities [41]. The architecture of ShuffleNetV2 is illustrated in Figure 6.

**Figure 7:**
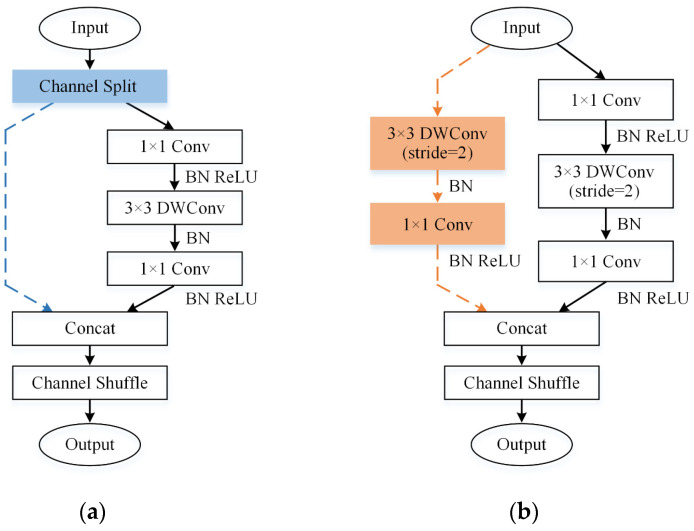
Two basic modules for ShuffleNetV2: (**a**) basic unit; (**b**) down-sampling unit.

From Figure 6, it is evident that the ShuffleNetV2 network consists of two Convolutional (Conv) modules and three Stages. The Conv module performs dimension reduction using convolution, batch normalization (BN), ReLU activation, and pooling layers. The reduced-dimensional features are sequentially fed into the three Stages to extract vibration signal characteristics further. Each Stage consists of two units called the basic unit and the down-sampling unit, as illustrated in Figure 7.

In the ShuffleNetV2 basic unit, the optimized time-frequency graphs are split into two branches. One branch preserves the original feature information. The other branch undergoes sequential operations of 1 × 1 regular convolution, 3 × 3 depth-wise convolution (DWConv), and another 1 × 1 regular convolution, all with a stride of 1 and the same number of input and output channels, ensuring the dimensions are unchanged. After convolution, the outputs of the two branches are concatenated and subjected to channel shuffling, effectively integrating vibration signal features across different channels.

In the down-sampling unit of ShuffleNetV2, the optimized time-frequency graphs are directly separated into two branches without channel split operations. Each branch includes a 2-stride DWConv with double output channels and half input dimensions. This enhances the computational efficiency of the network model while preserving essential vibration signal features.

### 4.2. ShuffleNetV2 Fault Diagnosis Framework

**Figure 8:**
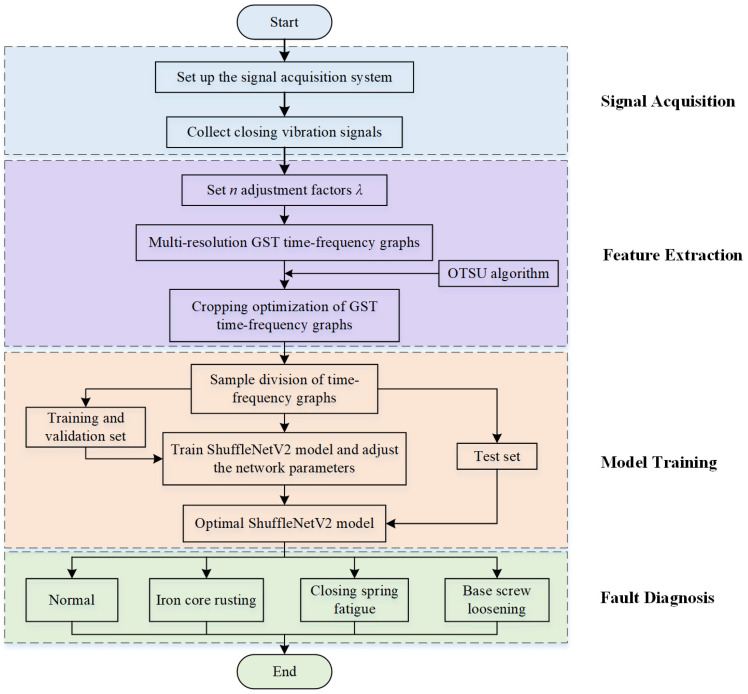
The framework of vacuum contactor fault diagnosis.

Figure 8 depicts the fault diagnosis framework for vacuum contactors, which consists of four parts: signal acquisition, feature extraction, model training, and fault diagnosis.

(1) Signal acquisition: A signal acquisition system is constructed according to the vacuum contactor fault simulation plan. Acceleration sensors are used to capture vibration signals during different states when the vacuum contactors close.
(2) Feature extraction: A series of multi-resolution GST time-frequency graphs of vibration signals are generated by combining Gaussian window width adjustment factors. The OTSU algorithm is employed to crop energy-concentrated regions from the GST time-frequency graphs.
(3) Model training: The optimized GST time-frequency graphs are partitioned into training, validation, and test sets. Model parameters are optimized, and the optimal ShuffleNetV2 model is obtained.
(4) Fault diagnosis: the optimal ShuffleNetV2 model is utilized to classify fault categories and output diagnostic outcomes.

## 5. Example Analysis

### 5.1. Time-Frequency Graph Dataset

In this study, GST time-frequency graphs of the closing vibration signals in four states are employed as signal features. The vibration signals in each state are augmented, and each state’s samples are expanded to 960. The dataset is randomly partitioned into training, validation, and test sets in a ratio of 6:2:2 for the same state, as detailed in Table 3.

### 5.2. Optimization Results of Time-Frequency Graph

**Figure 9:**
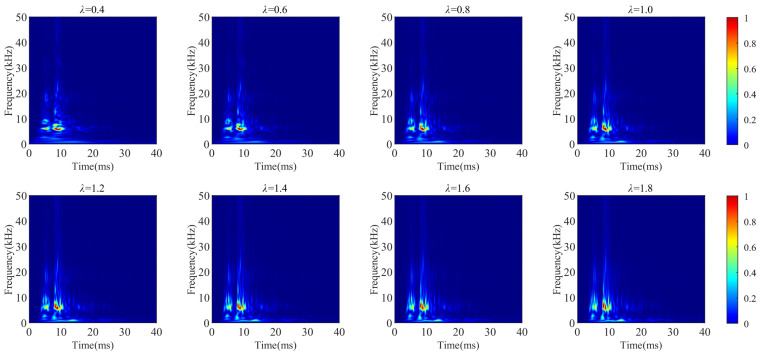
Multi-resolution GST time-frequency graphs.

A sample from the spring fatigue is selected. Eight sets of adjustment factors are applied to the GST expression to generate multi-resolution GST time-frequency graphs, as shown in Figure 9.

From Figure 9, it is observed that as the adjustment factor *λ* increases, the frequency resolution of the time-frequency graph is gradually improved from low-frequency bands to high-frequency bands. The characteristics of vibration signals are adequately represented across all frequency bands.

**Figure 10:**
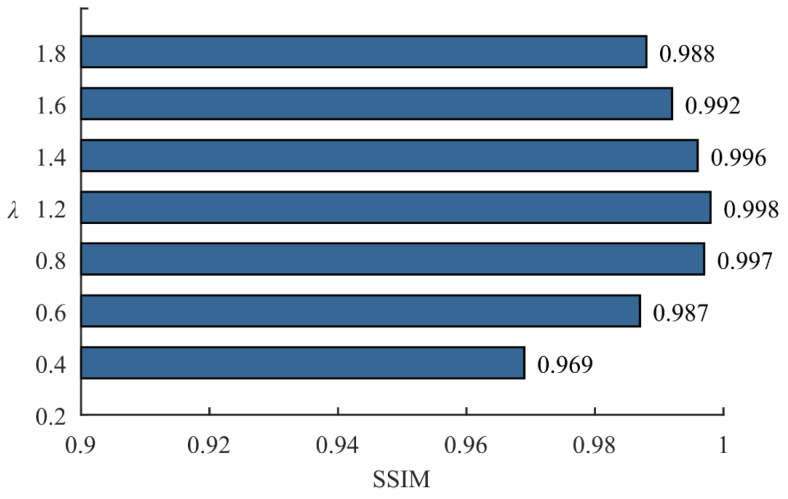
The values of SSIM between time-frequency graph augmented data and original data.

After data augmentation of the time-frequency graphs, the effect is evaluated using the structural similarity index measure (SSIM) [42]. SSIM is utilized to assess the similarity among images, with a range from −1 to 1. A higher SSIM value indicates greater similarity between the augmented time-frequency graphs and the original data. The SSIM calculation results are illustrated in Figure 10.

From Figure 10, it is evident that the SSIM values are all higher than 0.9. On one hand, this indicates a high level of consistency between the augmented time-frequency graphs and the original data, preserving important feature information of the original graphs. On the other hand, the augmentation of time-frequency graphs improves the diversity of the dataset.

**Figure 11:**
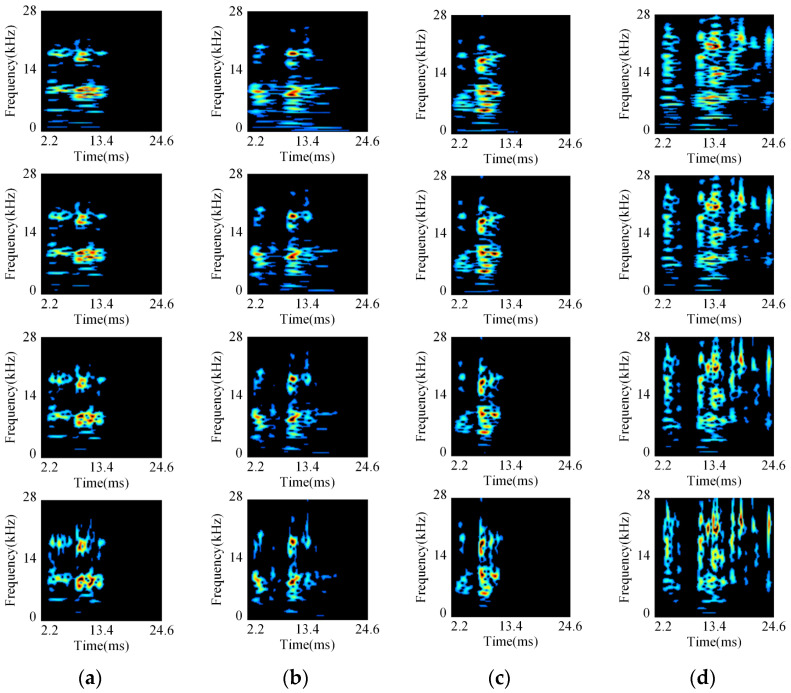
Optimization results of cropped GST time-frequency graphs: (**a**) normal; (**b**) iron core rusting; (**c**) closing spring fatigue; (**d**) base screw loosening.

The initial size of the GST time-frequency graphs is 224 × 224 pixels. After data augmentation and crop optimization rules given in Section 3.2, the size of the GST time-frequency graphs for each state is determined to be 125 × 125 pixels. Part of the crop optimization results are illustrated in Figure 11. The fault characteristics are highlighted, and the proportion of redundant pixels is significantly reduced, which is helpful in fault diagnosis accuracy and fast computational speed.

### 5.3. Performance Analysis of the Proposed Method

Based on the optimization results of the GST time-frequency graphs, the ShuffleNetV2 network is constructed on the Python 3.9 platform in the PyTorch environment. The specific network architecture is detailed in Table 4.

To enhance model performance, this study sets the initial learning rate of ShuffleNetV2 to 5 × 10^−4^ and the batch size to 32. Transfer learning is employed to load pre-trained models so as to accelerate network convergence. With fewer iterations, higher diagnostic accuracy is achieved, so the iteration count is set to 100.

**Figure 12:**
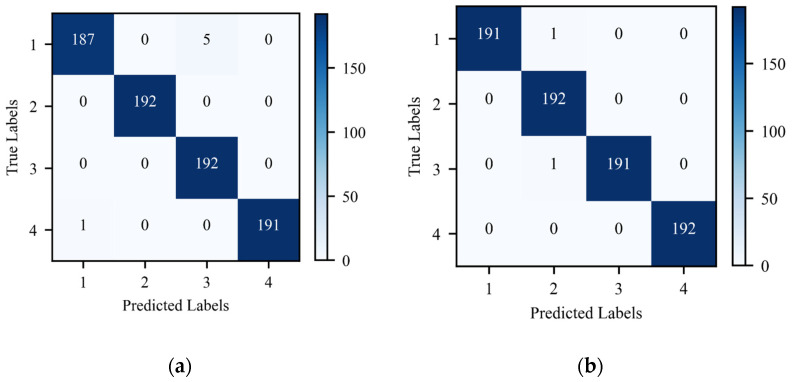
State recognition confusion matrices for GST time-frequency graphs: (**a**) without cropping optimization; (**b**) with cropping optimization.

The training sets of GST time-frequency graphs without and with cropping optimization are input into the ShuffleNetV2 networks to train the model. Then, the well-trained ShuffleNetV2 models are used to classify 192 test sets. The confusion matrices for state recognition of the test sets are shown in Figure 12a,b.

From Figure 12a, it is observed that in the GST time-frequency graphs without cropping optimization, the overall accuracy is 99.22%. The recognition accuracy for fatigue failure of springs and iron core rusting reaches 100%. There is one misclassified sample for base screw loosening fault, and in the normal state test set, five samples are misclassified as closing spring fatigue fault.

From Figure 12b, it is noted that in the GST time-frequency graphs with cropping optimization, the recognition accuracy of the test set reaches 99.74%. There is only one misclassified sample each for the normal state and closing spring fatigue fault. The accuracy is further improved.

The training and recognition times are shown in Table 5. Table 5 indicates that with the removal of the vast redundant background pixels during the graph optimization process, computational efficiency has been improved. The total training time is reduced by 9.24 min, and the total recognition time on the test set is decreased by 2.56 s. It is evident that the cropping optimization technique combined with the ShuffleNetV2 model maintains high diagnostic accuracy with finite computational resources.

### 5.4. Comparison of Different Network Structures

To validate the performance of the ShuffleNetV2 fault diagnosis model, the optimized vibration signal GST time-frequency graphs are input to the AlexNet, ResNet50, and ResNeXt50 networks to train. The training is set to 100 iterations, with a learning rate of 5 × 10^−4^ and a batch size of 32.

**Figure 13:**
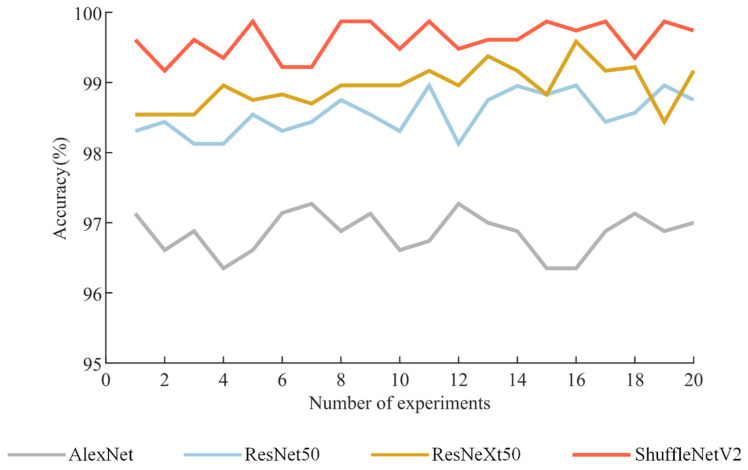
The accuracy of the test set for GST time-frequency graphs in four networks. AlexNet [28], ResNet50 [29], ResNeXt50 [30], ShuffleNetV2 [33].

All networks load pre-trained models to achieve optimal training results. The experiment is repeated 20 times, and the recognition accuracy of each network model is recorded, as shown in Figure 13.

According to Figure 13, the average test accuracy of the ShuffleNetV2 network reaches 99.61%, which is 2.36%, 1.05%, and 0.67% higher than those of the AlexNet, ResNet50, and ResNeXt50 networks. Moreover, the accuracy remains above 99% with minor fluctuations in 20 experiments, indicating its stable performance. Benefiting from the deeper networks, ResNet50 and ResNeXt50 networks can maintain high accuracy. However, due to the limited convolutional and pooling layers, the feature learning capability of AlexNet is constrained, which impacts its accuracy.

The average time for each iteration is shown in Table 6. It is evident that with channel split and channel shuffle structures, among the single iteration time of model training, ShuffleNetV2 is the fastest. Compared with AlexNet, ResNet50 and ResNeXt50, ShuffleNetV2 can reduce running time by up to 14%. AlexNet also runs faster than ResNet50 and ResNeXt50 because of its fewer layers. In addition, with cropping optimization, the time consumption is reduced obviously, especially in the case of ResNet50 and ResNeXt50, which reduce time consumption by 21.04% and 25.35%, individually, higher than 19.42% of ShuffleNetV2. This is because their networks are deeper and more sensitive to the scale of the input data. ShuffleNetV2 demonstrates significant advantages in both speed and accuracy for processing time-frequency graphs.

## 6. Conclusions

In this paper, the vacuum contactor closing vibration signals are transformed from one-dimensional time-series to two-dimensional time-frequency graphs using GST, combined with graph optimization techniques and the ShuffleNetV2 network, to be aware of the health state of the vacuum contactor. The following conclusions are drawn:

(1) GST introduces the Gaussian window width adjustment factor to generate multiple-resolution GST time-frequency graphs. This data augmentation technique ensures the extraction of overall time-frequency features of vibration signals, increases the diversity of the training dataset, and mitigates overfitting issues.
(2) The OTSU algorithm crops the energy concentration area of the GST time-frequency graphs. This process reduces 68.86% of redundant background pixels in these graphs. Therefore, the effective feature information of the critical frequency bands is kept, and size optimization is achieved simultaneously.
(3) A comparison is made between the AlexNet, ResNet50, ResNeXt50, and ShuffleNetV2 networks. ShuffleNetV2 can achieve the highest mean accuracy of 99.74%, and the single iteration time of model training is reduced by 19.42%.

In the future, we intend to explore regular updates for this fault diagnosis model to maintain high accuracy with more available operation data. We also aim to test the validity of the proposed model in other fields.

## Figures and Tables

**Table 2:** Vacuum contactor fault simulation scheme.

Status Category	Analogue Method	Collection Period	Number of Acquisitions
Normal	——	After industrial trials	50
		The first overhaul	40
		The second overhaul	30
Iron core rusting	A few iron filings added inside the core	After industrial trials	50
		The first overhaul	40
		The second overhaul	30
Closing spring fatigue	Spring pre-compression reduced by 3 mm	After industrial trials	50
		The first overhaul	40
		The second overhaul	30
Base screw loosening	Base screws screwed outwards 4 mm	After industrial trials	50
		The first overhaul	40
		The second overhaul	30

**Table 3:** Sample division of GST time-frequency graphs.

State	Label	Training Set Samples	Validation Set Samples	Test Set Samples
Normal	1	576	192	192
Iron core rusting	2	576	192	192
Closing spring fatigue	3	576	192	192
Base screw loosening	4	576	192	192

**Table 4:** Structure of the ShuffleNetV2 network model.

Model	Layer	Output Size	Kernel Size
ShuffleNetV2 fault diagnosis model	Input	125 × 125 × 3	
	Conv1	63 × 63 × 24	3 × 3
	MaxPool	32 × 32 × 24	3 × 3
	Stage2	16 × 16 × 116	
	Stage3	8 × 8 × 232	
	Stage4	4 × 4 × 464	
	Conv5	4 × 4 × 1024	1 × 1
	GlobalPool	1024	
	FC	4	

**Table 5:** Training and recognition time for GST time-frequency graph model without and with cropping optimization.

	Without Cropping Optimization	With Cropping Optimization
Model training time (min)	47.65	38.41
Total recognition time of the test set (s)	6.79	4.23

**Table 6:** The average time required for each iteration with different networks.

Network	Without Cropping Optimization	With Cropping Optimization
AlexNet [28]	33.26 s	27.32 s
ResNet50 [29]	39.49 s	31.18 s
ResNeXt50 [30]	43.99 s	32.84 s
ShuffleNetV2 [33]	28.43 s	22.91 s

## Data Availability

The data that support the findings of this study are available upon reasonable request from the corresponding author.

## Abbreviations

BN	Batch Normalization
BPNN	Back Propagation Neural Network
Conv modules	Convolutional modules
CWT	Continuous Wavelet Transform
DWConv	Depth-Wise Convolution
FC layer	Fully Connected layer
GST	Generalized Stockwell Transform
RF	Random Forest
SSIM	Structural Similarity Index Measure
ST	Stockwell Transform
STFT	Short-Time Fourier Transform
SVM	Support Vector Machine
